# Commentary on ‘Dissecting the hemagglutinin head and stalk specific IgG antibody response in healthcare workers following pandemic H1N1 vaccination’

**DOI:** 10.1038/npjvaccines.2016.4

**Published:** 2016-07-28

**Authors:** Ted M Ross

**Affiliations:** 1 Center for Vaccines and Immunology, University of Georgia, Athens, GA, USA

There is an urgent unmet need for more broadly protective seasonal influenza vaccines with greater breadth, enhanced potency, and durability of haemagglutination inhibition (HAI) responses. Currently licensed influenza vaccines induce HAI antibodies directed against the hemagglutinin (HA) head region that are largely strain-specific, and consequently do not cross-react with influenza strains that have undergone genetic drift. The elicitation of antibodies directed against the conserved stalk region of HA is an alternative approach to current head-based vaccines, and several stalk-based immunogens and vaccination strategies have been evaluated in pre-clinical models.^1–10^ In the study by Tete *et al.*^11^ presented in this issue of *npj Vaccines*, the authors describe the assessment of head and stalk antibodies in polyclonal sera obtained from health-care workers (HCW) following vaccination with a monovalent pandemic H1N1 influenza vaccine (Pandemrix; produced by GlaxoSmithKline) shortly after the pandemic H1N1 outbreak in October 2009. The HCW were divided into two categories, low or high HAI responders, based on their peak HAI response at day 21 and the longevity of the vaccine-induced response at day 90 post vaccination to the pandemic H1N1 strain. Collected serum samples were assessed for head- and stalk-directed responses. Compared to the head-directed antibody response, the stalk response comprised higher avidity, neutralizing antibodies following the initial vaccination of low responders. In addition, these stalk antibodies mediated antibody-dependent cellular cytotoxicity (ADCC) activity. The authors compared head and stalk antibody avidities and revealed that stalk-specific antibodies were qualitatively superior. Furthermore, stalk-specific antibodies mediated virus neutralization and had significantly higher ADCC activity than head-specific antibodies. Despite the reduced HAI titres in low responders, these individuals exhibited comparable antibody avidity, ADCC functionality, and neutralizing capacity to those of controls who had high HAI titres post vaccination.

These findings have implications for the development of a universal influenza vaccine approach. Universal vaccines have the potential to be paradigm-shifting for influenza vaccine landscape, with the goal of replacing seasonal vaccines with universal one(s). The pandemic H1N1 strain has an antigenically distinct head region compared to the HA, expressed by seasonal H1N1 influenza strains proceeding the 2009 pandemic.^12^ The introduction of this novel HA head region was the most likely reason for the preferential elicitation of stalk-specific antibodies, because secondary immunization of low responders preferentially boosted antibody titres directed against the head instead of the stalk. In addition, high responding individuals most likely possessed pre-existing memory B cells that were efficiently recalled to produce head-specific HAI antibodies. Previous studies indicate that chimeric HA antigens, in which the globular head is swapped out with the equivalent region from an exotic subtype, can elicit and boost stalk antibodies. Additionally, recently described immunogens elicited antibody responses directed against the stalk region.^2,9^ These strategies have proven to be effective in mice and ferret models,^1,3–8^ but have yet to be evaluated in humans. However, it was also demonstrated that sequential infection of ferrets with antigenically distinct seasonal H1N1 viruses could also elicit stalk-specific antibodies,^12^ therefore giving hope that these more broadly protective antibodies can be elicited using a variety of approaches.

The induction of ADCC activity by HA stalk-specific antibodies is one of the more important findings by Tete *et al*.^11^ ADCC assays assess the potential of antibodies, bound to FcγR of natural killer cells, to induce degranulation and release of cytokine (interferon-gamma) in the presence of a specific target antigen. Fc-FcγR interactions are required for HA stalk-specific antibody-mediated protection *in vivo*, supporting the importance of ADCC.^13–15^ Collectively, the increased affinity and functional ADCC activity by HA stalk-specific antibodies from low responders suggests that vaccination of these individuals conferred protection independent of modulating HAI activity.

Despite the increased appreciation for HA stalk-specific antibodies, there are still challenges to convert this knowledge into a long-term universal influenza vaccine strategy. First, the induction of stalk-specific antibodies will need to be achieved in the pre-immune human population. Unless the antigen is significantly different relative to the HA proteins/epitopes that established pre-existing memory, recalled B cells will likely mount an anti-head-biased response. The use of chimeric HA or stalk-specific immunogens administered two or three times in pre-clinical models show that stalk-specific antibodies can be induced, but whether these responses can be induced in people with pre-existing immune responses remains to be determined. In addition, there are little data on these approaches in animal models with pre-existing anti-influenza antibodies. Another aspect of a universal influenza vaccine approach is the induction of long-lived immunity in different populations. It is not clear if stalk-based vaccine strategies can elicit these long-lived memory B cells in humans, and whether these stalk-specific B cells would be preferentially recalled by subsequent vaccination. As these innovative vaccine strategies move forward into human clinical trials, it will be critical to establish that the phenomenon observed in this report during the change from two antigenically distinct H1N1 era can be recapitulated. Overall, this report by Tete *et al.*^11^ is a fascinating look into the induction of HA stalk-specific antibodies and how these antibodies would work in humans.
